# Primed primary metabolism in systemic leaves: a functional systems analysis

**DOI:** 10.1038/s41598-017-18397-5

**Published:** 2018-01-09

**Authors:** Jens Schwachtje, Axel Fischer, Alexander Erban, Joachim Kopka

**Affiliations:** Department of Molecular Physiology: Applied Metabolome Analysis, Max-Planck-Institute of Molecular Plant Physiology, Am Mühlenberg 1, D-14476 Potsdam-Golm, Germany

## Abstract

Plants evolved mechanisms to counteract bacterial infection by preparing yet uninfected systemic tissues for an enhanced defense response, so-called systemic acquired resistance or priming responses. Primed leaves express a wide range of genes that enhance the defense response once an infection takes place. While hormone-driven defense signalling and defensive metabolites have been well studied, less focus has been set on the reorganization of primary metabolism in systemic leaves. Since primary metabolism plays an essential role during defense to provide energy and chemical building blocks, we investigated changes in primary metabolism at RNA and metabolite levels in systemic leaves of *Arabidopsis thaliana* plants that were locally infected with *Pseudomonas syringae*. Known defense genes were still activated 3–4 days after infection. Also primary metabolism was significantly altered. Nitrogen (N)-metabolism and content of amino acids and other N-containing metabolites were significantly reduced, whereas the organic acids fumarate and malate were strongly increased. We suggest that reduction of N-metabolites in systemic leaves primes defense against bacterial infection by reducing the nutritional value of systemic tissue. Increased organic acids serve as quickly available metabolic resources of energy and carbon-building blocks for the production of defense metabolites during subsequent secondary infections.

## Introduction

During long-term co-evolution with pathogenic bacteria, plants gained the ability to prepare yet non-infected (systemic) tissue for enhanced (primed) defense responses once bacteria attacked locally other parts of the plant^1,2^. This mechanism is known as systemic acquired resistance (SAR). SAR is induced after the plant either recognized certain pathogen-associated molecular patterns (PAMPs), such as flagellin and lipopolysaccharides for PAMP-triggerd immunity (PTI), or effector proteins that are introduced into host cells by the pathogen’s Type III secretion system to suppress innate immunity for effector-triggered immunity (ETI)^3–5^. Both PTI and ETI induce partially overlapping defense responses in infected leaves of *Arabidopsis thaliana* ^6,7^. These defenses impose physiological costs due to activation of signal cascades, production of defense metabolites and general re-organization of primary metabolism^8,9^. These metabolic changes lead to loss of energy and resources that would otherwise be available for growth and reproduction under non-stressed conditions^10^. For example, after a local pathogen attack, the plant’s photosynthetic capacity is drastically impaired by down-regulation of genes of the 2 photosystems. In addition stomata close and form a physical barrier against bacterial entry into the leaf at the trade-off of limited CO_2_ supply^11,12^. Amino acid pools are strongly reduced in locally infected leaves already several hours after the infection^13^. It is largely unknown, which metabolic changes are necessary for fuelling the plant’s defense response and which changes are actively manipulated by the pathogens in order to better access to essential nutrients^14,15^. Furthermore, Schwachtje and Baldwin^16^ suggested that re-modelling of primary metabolism in its own right may act as a defense component.

*Pseudomonas syringae* is a pathogen with a hemi-biotrophic life-style and exclusively depends on plant metabolites for its propagation. During the early phase of the infection, *P. syringae* colonizes the apoplast and is well adapted to utilize nutrients present in the apoplastic fluid^17^. Several strains of *P. syringae* lack genes that are required for uptake and catabolism of metabolites that are typically present at low concentrations in the apoplast, such as valine, isoleucine or glyoxylate^14,18,19^. These strains can still efficiently metabolize abundant metabolites as glutamate, glutamine, aspartate, asparagine and GABA^20^. Abundant metabolites have been shown to increase locally in leaves early after an infection^13^. We therefore argue that the pathogen’s capability to utilize such nutrients is a specific adaptive mechanism and integral part of bacterial infection.

It is suggested that bacteria developed the ability to actively interfere with plant metabolism and defense via effector proteins and different other compounds^15^. For example, *P. syringae* produces coronatine, which mimics the hormone conjugate jasmonic acid-isoleucine and may negatively interfere with salicylic acid signalling, which regulates anti-bacterial defenses in plants^21^. Certain lipopeptides are produced by *P. syringae* pathogenic varieties, which alter integrity of cell membranes^22^. Other pathovars of *P. syringae* produce so-called antimetabolites, which are small peptide-like molecules that are thought to interfere with enzymes of nitrogen metabolism^23–25^, such as glutamine synthase and ornithine-metabolizing enzymes. Recently, different mechanisms of competition for apoplastic sugars between the plant and pathogens have been elucidated. For example, in response to *Pseudomonas syringae*, Arabidopsis enhances the activity of a monosaccharide transporter that removes sugars from the apoplast^26^. *Phytophtora sojae* produces xyloglucan endoglucanases that release monosaccharides from the cell wall, which is counteracted by glucanase inhibitors from soybean^27^. Therefore, we expect that it would be advantageous for the plant to modify primary metabolism of yet non-infected leaves to reduce the nutritional quality of the apoplastic fluid and to prepare leaves so that the influence of pathogens on metabolism is counteracted.

In this study we explore, how plant primary metabolism in systemic tissue changes after local infection with bacterial pathogens and how these changes differ from known local metabolic changes in infected tissues. We investigate both, transcriptional and metabolic medium-term responses of yet non-infected systemic leaves 3 to 4 days after a first localized infection of leaves with avirulent *P. syringae* avrRpm1. In a systems biology approach, we determine the main genes that are responsible for re-organization of primary metabolism and correlate transcript changes with relative changes of primary metabolites. We discuss how alterations of tricarboxylic acid (TCA)-cycle, amino acid and sugar metabolism may serve as integrated mechanisms of primed plant defense responses.

## Methods

### Plants and bacteria

*Arabidopsis* Col-0 plants were germinated on half-strength MS medium. After 10 days plants were individually transferred to single pots with peat substrate. Plants were grown in a growth chamber with a 16 h light phase at 150 µmol m^−2^s^−2^, 20 °C at night, 22 °C during the day, and relative air humidity of 70%. 4 week old plants were used for the experiment. Three lower mature leaves per plant were inoculated 5 h before the end of the light phase, i.e. 11 h after light on (dawn), by syringe-infiltration with ~15 µl of *P. syringae* avrRpm1 (*Pst*) in 10 mM MgCl_2_ at OD_600_ = 0.02 (10^7^ cfu/ml), mock treatment consisted of 10 mM MgCl_2_.

### Profiling of the primary metabolome

**Figure 1:**
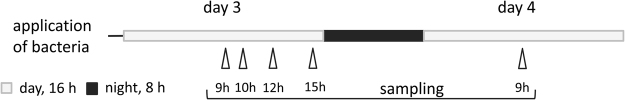
Scheme of harvesting time points. Samples were taken at 5 time points over 2 days, each shown as hours after dawn. Metabolites were measured at all time points, RNA-Seq was carried out at 10 h and 15 h during day 3 and at 9 h during day 4. Plants were inoculated or mock treated at 11 h after dawn.

At day 3 and 4 after infection or mock infection young systemic leaves were harvested and snap frozen in liquid nitrogen. The sampling times were at 9 h, 10 h, 12 h, 15 h and 9 h after dawn, respectively, of the 16 h day (Fig. 1). Four to 7 independent replicates per treatment and time point were analysed. Profiling of primary metabolites was performed as described by Erban *et al*.^28^. Frozen leaf tissue (40–100 mg) was ground (2 × 45 s, maximum frequency) by a Retsch mill (MM 400, Retsch, Haan, Germany). Per mg of leaf tissue, metabolites were extracted first with 4.5 µl methanol containing 0.2 mg*mL^−1^ U-^13^C-sorbitol as internal standard at 70 °C for 15 min and subsequently with 2.5 µL chloroform for 5 min at 37 °C. The liquid was partitioned by adding 5 µL H_2_O per mg leaf tissue to obtain a fraction enriched with polar metabolites. The polar phase (~160 µL) was completely dried in a vacuum concentrator. The polar fraction was derivatized by methoxyamination and trimethylsilylation. A mixture of n-alkanes (C_12_, C_15_, C_18_, C_19_, C_22_, C_28_, C_32_ and C_36_) served as retention index standards^29^. A 1 µL aliquot of the samples was injected in splitless mode at 230 °C into a 6890N24 gas chromatograph (Agilent Technologies, Böblingen, Germany; http://www.agilent.com). The sample was separated on a Varian FactorFour column (VF-5 ms, length 30 m, diameter 0.25 mm, and 0.25 µm film thickness (Agilent Technologies, Böblingen, Germany) using the following temperature programme 1 min at 70 °C; ramp to 350 °C at 9°/min, 5 min at 350 °C, then cooling. Compounds were detected by electron ionization/time-of-flight mass spectrometry (EI-TOF-MS) using a Pegasus III TOF mass spectrometer (LECO Instrumente GmbH, Möchengladbach, Germany). Chromatograms were obtained and baseline corrected by ChromaTOF software (Version 4.22, LECO, St. Joseph, USA). Identification of metabolites was manually supervised with the TagFinder software^30^ and the mass spectra and retention time index (RI) reference collection of the Golm Metabolome Database^31,32^. Peak heights were normalized to U-^13^C-sorbitol and fresh weights.

### RNA sequencing (RNA-Seq) analysis

Leaf harvests at day 3 were done at 10 h and 15 h after dawn and at day 4 at 9 h after dawn. Frozen leaf tissue (40–100 mg) from three independent replicates each of bacteria-primed and mock treated was ground (2 × 45 s, maximum frequency) by a Retsch mill (MM 400, Retsch, Haan, Germany). Each harvest was a young systemic leaf from a single rosette. The harvested systemic leaves were all developmentally younger than the previously locally infected leaves. RNA was extracted with the RNeasy Plant Mini Kit according to the manual (QIAGEN GmbH, Hilden, Germany). RNA was quantified with a Qubit® RNA HS Assay Kit (ThermoFisher Scientific Life Technologies GmbH, Darmstadt, Germany) and RNA quality was further assessed by agarose gel electrophoresis. Sequencing was carried out at the Max-Planck-Genome Centre (Cologne, Germany) including quality check by Agilent 2100 Bioanalyzer (Agilent Technologies GmbH & Co. KG, Waldbronn, Germany), polyA mRNA enrichment, RNA library preparation, and sequencing at 3 gigabase (Gb) raw reads per sample by Illumina HiSeq 3000 sequencers (Illumina Inc., San Diego, CA, USA). The expression data were uploaded to the Gene Expression Omnibus (GEO, https://www.ncbi.nlm.nih.gov/geo/) and made available through accession number GSE101839. Sequence data of all samples were mapped with STAR v2.5.2a using default parameters^33^. Ensembl version 31 (TAIR10) genome reference in FASTA format (ftp://ftp.ensemblgenomes.org/pub/release-31/plants/fasta/arabidopsis_thaliana/dna/Arabidopsis_thaliana.TAIR10.31.dna.toplevel.fa.gz) and Ensembl version 31 cDNA Annotation in GTF format (ftp://ftp.ensemblgenomes.org/pub/release-31/plants/gtf/arabidopsis_thaliana/Arabidopsis_thaliana.TAIR10.31.gtf.gz) were used for genome indexing. Anti-strand read counts from the ReadsPerGene files of all 18 samples were merged in order to perform a differential expression analysis with DSeq2^33,34^ as guided by the rnaseqGene Bioconductor workflow (https://www.bioconductor.org/help/workflows/rnaseqGene/). Briefly, samples were grouped by their three replicates, read count data were then loaded with DESeqDataSetFromMatrix to create a DeSeqDataSet object to subsequently run the standard analysis consisting of the functions DESeq and Results. Transcript abundance data were expressed as log_2_-transformed fold changes (log2fc) of mature primed versus mock-treated leaves from single plants. In all pairwise comparisons, genes were defined as differentially expressed if the Benjamini-Hochberg adjusted P-value was lower than 0.05. Gene set enrichment analysis was done with clusterProfile for KEGG terms and PlantGSEA for GO terms^35,36^. The significance threshold of gene set enrichments was false discovery rate (FDR) < 0.05.

## Results

### Bacteria- induced transcriptional changes in systemic leaves affect primary metabolic functions and are under circadian control

**Figure 2:**
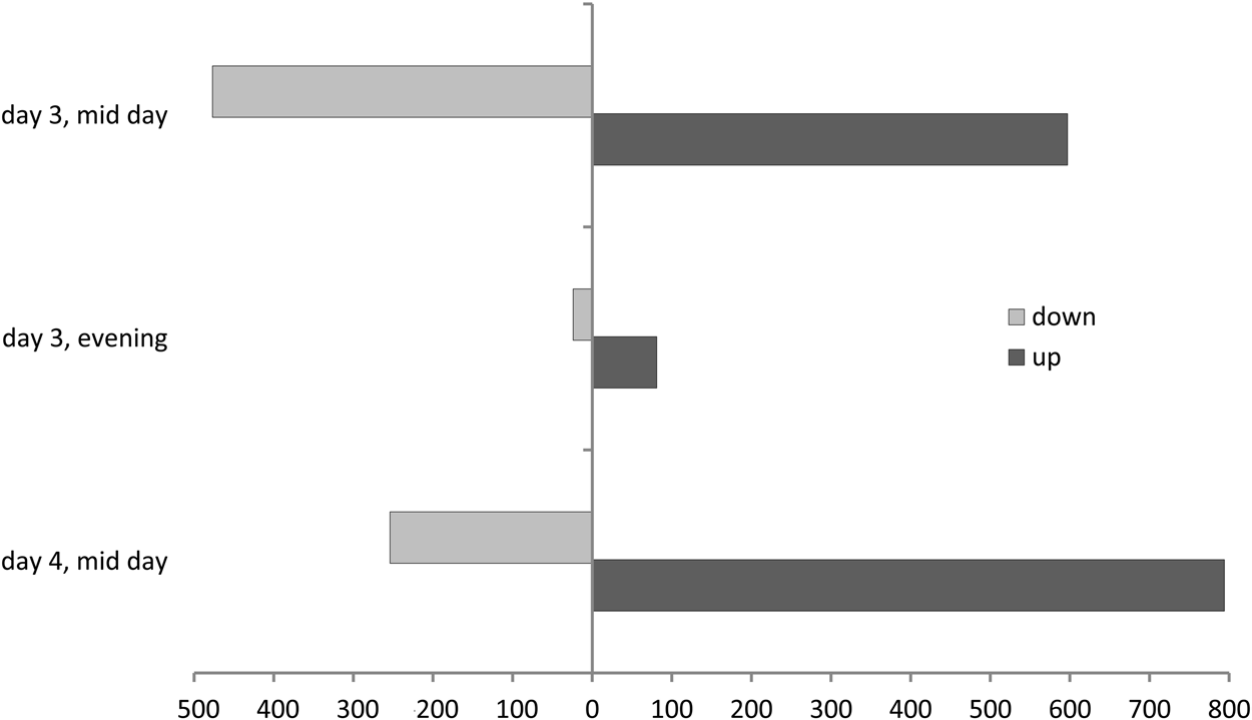
Overview of number of up- and down-regulated genes at each harvest time point.

For the investigation of the systemic transcriptional response of mature systemic leaves to a bacterial infection we selected a period of 3–4 days after *Pst* infection. The return of most induced responses was thought previously to be one of the basic requirements for the study of the relevant plant priming and memory phenomena^37^. At day three after priming, 10 h after dawn, 597 differentially expressed genes relative to the mock treatment were upregulated and 477 genes were downregulated (Fig. 2). The major upregulated gene sets were related to biotic stress response functions (Table 1, Supplementary Table S1), such as the gene ontology (GO) defined gene sets, defense response (GO: 0006952, FDR: 2.11E-3), salicylic acid biosynthetic process (GO: 0009697, FDR: 1.07E-17) and systemic acquired resistance (GO: 0009627, FDR: 5.3E-45), including PR1 (At2g14610; lg2fc at day 3, 15 h: 2.92, lg2fc at day 4: 2.84). These observation confirmed successful bacterial infection of the plant system. Several gene sets related to primary metabolism were downregulated, such as the cellular carbohydrate metabolic process (GO: 0044262, FDR: 3.59E-15), cellular nitrogen compound catabolic process (GO: 0044270, FDR: 4.73E-9) and generation of precursor metabolites and energy (GO: 0006091, FDR: 2.35E−23). These results indicated medium-term highly significant re-organization of primary metabolism in systemic leaves that was associated with anti-bacterial plant defense responses. These processes were furthermore accompanied by the upregulation of the gene set carboxylic acid biosynthetic process (GO: 0046394, FDR: 4.31E-3). Also, photosynthesis-related genes were downregulated, namely light reaction (GO: 0019684, FDR: 3.27E-12), photosystem II assembly (GO: 0010207, FDR: 8.74E-5) and chlorophyll metabolic process (GO: 0015994, FDR: 1.21E-11).

**Table 1:** List of significantly regulated gene sets, treatment vs. mock for three time points (days – hour after dawn).

Gene Set Name (number of genes in set)	Day 3, 10 h		Day 3, 15 h		Day 4, 9 h		GO number
	Number of regulated genes	FDR of GO term vs. mock treatment	Number of regulated genes	FDR of GO term vs. mock treatment	Number of regulated genes	FDR of GO term vs. mock treatment	
DEFENSE_RESPONSE (1644)	129	**2.11E-34**	33	**7.52E-17**	88	**7.22E-07**	GO: 0006952
SYSTEMIC_ACQUIRED_RESISTANCE (444)	83	**5.30E-45**	22	**7.96E-18**	52	**9.32E-15**	GO: 0009627
DEFENSE_RESPONSE,_INCOMPATIBLE_INTERACTION (535)	92	**2.60E-47**	24	**1.81E-18**	58	**3.77E-15**	GO: 0009814
SALICYLIC_ACID_BIOSYNTHETIC_PROCESS (209)	36	**1.07E-17**	13	**1.79E-11**	27	**3.10E-08**	GO: 0009697
CELLULAR_CARBOHYDRATE_METABOLIC_PROCESS (1771)	86	3.59E-15	—	n.s.	—	n.s.	GO: 0044262
CELLULAR_NITROGEN_COMPOUND_CATABOLIC_PROCESS^88^	16	4.73E-09	—	n.s.	11	2.28E-06	GO: 0044270
GENERATION_OF_PRECURSOR_METABOLITES_AND_ENERGY (730)	63	2.35E-21	—	n.s.	—	n.s.	GO: 0006091
CARBOXYLIC_ACID_BIOSYNTHETIC_PROCESS (1113)	44	**4.31E-03**	13	**8.19E-04**	—	n.s.	GO: 0046394
PHOTOSYNTHESIS,_LIGHT_REACTION (333)	33	3.27E-12	—	**n.s**.		n.s.	GO: 0019684
PHOTOSYSTEM_II_ASSEMBLY (177)	15	8.74E-05	—	**n.s**.	—	n.s.	GO: 0010207
CHLOROPHYLL_METABOLIC_PROCESS (188)	25	1.21E-11	—	**n.s**.	12	3.05E-04	GO: 0015994
RNA_METHYLATION (185)	35	**2.94E-18**	—	**n.s**.	127	**2.76E-112**	GO: 0001510
RIBOSOME_BIOGENESIS (435)	33	**9.88E-08**	—	**n.s**.	118	**5.06E-68**	GO: 0042254
PYRIMIDINE_RIBONUCLEOTIDE_BIOSYNTHETIC_PROCESS (140)	22	**4.43E-10**	—	**n.s**.	72	**6.94E-56**	GO: 0009220
RESPONSE_TO_OXIDATIVE_STRESS (586)	30	2.00E-05	—	n.s.	—	n.s.	GO: 0006979
RESPONSE_TO_SALT_STRESS (780)	33	2.22E-04	—	n.s.	—	n.s.	GO: 0009651
RESPONSE_TO_OSMOTIC_STRESS (834)	34	3.33E-04	6	4.71E-02	—	n.s.	GO: 0006970
RESPONSE_TO_COLD (622)	27	1.10E-03	—	n.s.	—	n.s.	GO: 0009409

All shown GO sets are biological processes. FDR of upregulated gene sets are marked in bold font, downregulated in normal font, n.s.: not significant.

Subsequently, at the end of day 3 after priming (at 15 h of the light period, one hour before onset of the night), the number of significantly regulated genes was much less compared to the mid of the light phase (Fig. 2). Only 81 genes were upregulated and even less, i.e. 24, were downregulated. The number of remaining regulated genes was only 10% of the number of genes that were regulated at the 10 h time point of the same day. Most upregulated GO gene categories were related to defense response and signalling (Table 1, Supplementary Table S1). Photosynthesis-related gene GO sets were not regulated any longer and most of the sets related to primary metabolism that were regulated during the light phase were either not regulated or to a much lesser degree (Table 1, Supplementary Table S1).

At day 4 after priming, again approximately in the middle of the light phase, i.e. 9 h after dawn, the number of significantly regulated genes increased again to 794 up- and 254 downregulated genes (Fig. 2). The most upregulated gene sets were related to RNA metabolism, such as RNA methylation (GO: 0001510, FDR: 2.8E-112), ribosome biogenesis (GO: 0042254, FDR: 5.06E-68) and pyrimidine ribonucleotide biosynthetic process (GO: 0009220, FDR: 6.94E-56). Genes of the cellular nitrogen compound catabolic process (GO: 0044270, FDR: 2.28E-6) and the chlorophyll metabolic process (GO: 0015994, FDR: 3.05E-4) returned to be significantly changed. Still, the previously changed defense related gene sets were upregulated, specifically the defense response sub set incompatible interaction (GO: 0009814, FDR: 3.77E-15) and systemic acquired resistance (GO: 0009627, FDR: 9.32E-15).

The different RNA-Seq profiles at the three analysed time points and specifically the transient attenuation at the end of the day indicate that the priming response in systemic leaves is under strong circadian control. The reduced set of genes in the evening of day 3 did not contain genes related to primary metabolism. The overrepresented categories, however, were defense and signalling (Table 1, Supplementary Table S1). To further dissect this observation we analysed circadian clock related genes. The two core clock genes were slightly regulated at day three at 10 h, namely CCA1 (At2g46830, lg2fc -0.9) and LHY/CCA1-like 1 (At5g02840, lg2fc -0.6), but not at the other two time points (Table 2). This observation indicates that the strongly attenuated transcriptional regulation at the end of day 3 is likely not caused by a pathogen-induced shift of the circadian clock but rather under control of the normal circadian clock. This leads to a general reduction of a subset of systemically induced transcriptional activities shortly before the onset of the night.

**Table 2:** List of regulated genes at day 3 and day 4 in systemic leaves after treatment.

**TAIR ID**	**symbol**	log2fc day 3 10 h	log2fc day 3 15 h	log2fc day 4 9 h	**Gene name**	
AT3G47340	ASN1	−3.30		−1.93	glutamine-dependent asparagine synthase 1	N metabolism
AT2G39800	P5CS1	−2.07			delta1-pyrroline-5-carboxylate synthase 1	
AT5G17330	GAD	−1.83			glutamate decarboxylase	
AT5G04140	GLU1	−0.87			glutamate synthase 1	
AT5G09660	NAD-PMDH2	−0.82			peroxisomal NAD-malate dehydrogenase 2	
AT5G35630	GS2	−0.82			glutamine synthetase 2	
AT3G22200	POP2	−0.68		−0.63	pyridoxal phosphate (PLP)-dependent transferases superfamily protein	
AT3G55610	P5CS2	−0.62			delta 1-pyrroline-5-carboxylate synthase 2	
AT1G30120	PDH-E1 BETA	−0.44			pyruvate dehydrogenase E1 beta	
AT1G65960	GAD2	−0.36			glutamate decarboxylase 2	
AT1G04410	c-NAD-MDH1	−0.34			lactate/malate dehydrogenase family protein	
AT1G79750	NADP-ME4	−0.29		−0.28	NADP-malic enzyme 4	
AT1G59900	PDH-E1 ALPHA	−0.28			pyruvate dehydrogenase complex E1 alpha subunit	
AT4G13510	AMT1	0.84			ammonium transporter 1	
AT2G38290	AMT2	1.14			ammonium transporter 2	
AT5G50200	NRT3.1	1.39		1.42	nitrate transmembrane transporter	
AT1G08650	PPCK1	1.47		1.40	phosphoenolpyruvate carboxylase kinase 1	
AT5G45380	DUR3	2.41	2.26	1.75	urea-proton symporter DEGRADATION OF UREA 3 (DUR3)	
AT1G09350	GolS3	−1.61		−2.20	galactinol synthase 3	C metabolism
AT1G42970	GAPB	−0.97			glyceraldehyde-3-phosphate dehydrogenase B subunit	
AT1G12780	UGE1	−0.87			UDP-D-glucose/UDP-D-galactose 4-epimerase 1	
AT1G11720	SS3	−0.69			starch synthase 3	
AT3G12780	PGK1	−0.69			phosphoglycerate kinase 1	
AT1G74030	ENO1	−0.68			enolase 1	
AT4G24620	PGI1	−0.61			phosphoglucose isomerase 1	
AT5G19220	APL1	−0.61		−0.51	ADP glucose pyrophosphorylase large subunit 1	
AT2G35840	AT2G35840	−0.60			Sucrose-6F-phosphate phosphohydrolase family protein	
AT3G23920	BAM1	−0.55			beta-amylase 1	
AT5G22510	INV-E	−0.54		−0.52	alkaline/neutral invertase	
AT1G22710	SUC2	−0.40			sucrose-proton symporter 2	
AT1G23190	PGM3	−0.38			Phosphoglucomutase/phosphomannomutase family protein	
AT3G08590	iPGAM2	−0.31			Phosphoglycerate mutase, 2,3-bisphosphoglycerate-independent	
AT4G23010	UTR2	0.76			UDP-galactose transporter 2	
AT1G14360	UTR3	1.05			UDP-galactose transporter 3	
AT4G25000	AMY1	1.27		1.38	alpha-amylase-like protein	
AT2G02810	UTR1	1.30			UDP-galactose transporter 1	
AT2G26440	AT2G26440	1.58			plant invertase/pectin methylesterase inhibitor superfamily	
AT4G02330	ATPMEPCRB			1.70	plant invertase/pectin methylesterase inhibitor superfamily	
AT5G44420	PDF1.2	−4.1		2.9	plant defensin 1.2	jasmonic acid dependent
AT2G26020	PDF1.2b	−3.0			plant defensin 1.2b	
AT5G44430	PDF1.2c	−3.5			plant defensin 1.2C	
AT2G26010	PDF1.3	−3.5			plant defensin 1.3	
AT2G46830	CCA1	−0.9			circadian clock associated 1	circadian clock
AT5G02840	LCL1	−0.6			LHY/CCA1-like 1	
AT4G35770	SEN1	−2.4		−2.2	rhodanese/cell cycle control phosphatase superfamily protein	induced by sugar starvation
AT3G13450	BCKDH E1β	−0.5		−0.6	transketolase family protein	
AT1G03090	MCCA sub1	−0.9		−0.7	methylcrotonyl-CoA carboxylase alpha chain	
AT2G42540	COR15A	−2.4	−2.1	−1.7	cold-regulated 15a	cold acclimation
AT2G42530	COR15B	−1.7	−1.5	−1.6	cold-regulated 15b	
AT4G25480	DREB1A	−1.2		−1.5	dehydration response element B1A	

Log_2_-fold change at indicated days after inoculation and hour after dawn relative to controls is shown and all listed genes are significantly regulated (P < 0.05). Metabolism-, defense-, stress-, sugar starvation-, and clock-related genes are listed as indicated in the last column.

### Transcriptional changes include elements of abiotic stress responses

Gene sets associated with abiotic stresses were significantly downregulated three days after priming (Table 1, Supplementary Table S1), such as response to oxidative stress (GO: 0006979, FDR: 2.0E -5), response to salt stress (GO: 0009651, FDR: 2.22E -4), response to osmotic stress (GO: 0006970, FDR: 3.33E -4) and response to cold (GO: 0009409, FDR: 1.1E -3). Specifically, the marker genes for cold acclimation, Cor15A (At2g42540) and Cor15B (At2g42530), showed consistent down-regulation at all 3 time points. Also, DREB1A (At4g25480) and galactinol synthase 3 (At1g09350) were downregulated at day 3 and 4 during the day (Table 2). Moreover, senescence- and sugar starvation-associated genes, such as SEN1 (At4g35770), BCKDH E1β (At3g13450), MCCA sub1 (At1g03090) and ASN1 (At3g47340) were significantly downregulated during day 3 and 4 (Table 2).

### Priming induces persistent medium-term metabolic re-organization in systemic leaves

Defense responses do not only cause transcriptional reprogramming, activation of defense related signalling pathways and the production of defensive metabolites but also the re-organization of primary metabolism to supply energy and precursors^8–10^. We therefore investigated the metabolite profiles of primed leaves to unravel and further characterize the primed metabolic state. Metabolites were measured at 5 time points, 4 at day 3 and one time at day 4 (Fig. 1). The metabolites of primary metabolism were significantly changed in systemic leaves. We shortly report the primed metabolic changes grouped by major metabolite classes and will integrate and discuss these and the transcriptional responses in a detailed discussion.

Several amino acids and nitrogen containing compounds were reduced in systemic leaves (Table 3, Supplementary Table S2): serine, glycine, proline, glutamic acid, and putrescine were strongly reduced at day 3 and reached control or higher levels again at day 4. Ornithine, that in GC-MS based profiling due to technological constraints represents the sum of the urea-cycle intermediates arginine, citrulline and ornithine, was reduced at all 5 time points, with highest significance 9 h after dawn at day 3. Other amino acids, such as threonine and valine were not changed at day 3 but increased significantly at day 4. Leucine and alanine did not change significantly, but alanine tended to be downregulated at day 3 and was upregulated at day 4.

**Table 3:** List of regulated metabolites in systemic leaves at day 3 and day 4 (days – hour after dawn) after treatment.

Metabolite	Day 3				Day 4 fold change
	fold change				
	9 h	10 h	12 h	15 h	9 h
Alanine	nd	1.09	0.63	0.70	1.46
β-Alanine	**0.58**	**0.43**	0.75	**0.62**	1.13
Glutamic acid	nd	nd	**0.58**	**0.31**	1.43
Glycine	**0.44**	0.56	**0.47**	0.82	**1.96**
Leucine	nd	nd	0.83	0.99	nd
Ornithine	0.88	**0.51**	0.66	1.90	0.83
Serine	0.82	0.69	0.46	0.99	1.10
Proline	**0.20**	**0.13**	**0.37**	0.44	1.04
Putrescine	**0.29**	0.25	0.51	0.70	1.47
Threonine	nd	nd	0.85	0.85	**1.71**
Valine	nd	1.06	1.04	0.95	**1.67**
Fumaric acid	**22.86**	**9.43**	**15.11**	**4.03**	**6.62**
Glyceric acid	**1.71**	0.98	0.86	**0.60**	**2.13**
Glycolic acid	1.12	1.17	**1.25**	nd	**1.52**
Malic acid	**12.91**	**6.61**	**5.99**	**4.81**	**7.99**
Phosphoric acid	0.77	0.42	0.88	0.91	0.97
Shikimic acid	0.78	0.90	1.20	0.86	1.31
*trans-*Sinapic acid	**0.37**	0.61	0.9	0.51	0.82
Threonic acid	**2.16**	**2.09**	**1.79**	1.11	**1.58**
1,6-Anhydroglucose	**0.49**	0.61	1.06	0.82	0.89
Fructose-6-P	nd	nd	0.80	nd	1.34
Glucose	**0.22**	**0.20**	0.64	**0.44**	0.55
Glucose-6-P	nd	nd	0.70	nd	1.68
Maltose	nd	0.72	**1.55**	0.85	0.92
Sucrose	0.85	0.87	1.00	**0.79**	1.06

Fold-changes of metabolite levels relative to mock-infected controls are shown. Ornithine represents the sum of ornithine, citrulline and arginine. Significant metabolites are shown in bold (t-test; P < 0.05; n.d., not detectable).

The organic acids fumarate and malate showed large and highly significant upregulation during all time points (Table 3). Shikimic acid was upregulated at day 4 and *trans*-sinapic acid was downregulated at the first time point at day 3. Threonic acid was significantly upregulated during day 3 and 4, except for the last time point at day 3.

Glucose was significantly reduced at day 3 and remained reduced at day 4. Glucose-6-P and fructose-6-P were not significantly regulated and sucrose was only significantly regulated at the late time point at day 3, where it was slightly down.

## Discussion

We investigated the medium-term primed state of systemic leaves at 3 and 4 days after priming both at transcriptional and the metabolic levels. Marked changes in metabolites were in accordance with transcriptional changes in nitrogen and carbon metabolism. The primed re-organization of primary metabolism is likely a combined result of a) indirect, passive effects caused by the local infection on global metabolism of the plant, such as altered nutrient uptake by the root or reduced photosynthetic activity in infected leaves and b) can be affected by signals derived from infected leaves that induce re-organization of primary metabolism in systemic leaves. The latter implicates that the metabolic changes may serve for a better defense response once a potential pathogen infection occurs in the systemic leaves. The several possible implications of primed primary metabolites for an enhanced defense response are discussed in the following.

### Nitrogen limitation within systemic leaves

Several genes of core nitrogen metabolism were regulated in systemic leaves, mostly affecting amino acid metabolism. Especially genes of asparagine, glutamate and glutamine synthesis, the GABA shunt and transaminases were downregulated (Table 2, Supplementary Table S3). This indicates nitrogen limitation in systemic leaves that is associated with reduced availability of amino acids. Accordingly, the GO category “cellular response to nitrogen starvation” was significantly upregulated (Table 1, Supplementary Table S1) and, indeed, the metabolic levels of several proteinogenic and also non-proteinogenic amino acids are reduced in systemic leaves, such as proline, serine, glutamic acid, and beta-alanine (Table 3). Concomitantly, ammonium transporters AMT1 and 2, nitrate transporter NRT3.1 and the urea transporter DUR3 were upregulated (Table 2), which support N-uptake from the apoplast^38^. Taken together, these data indicate a mechanism by which primed systemic leaves are not only better prepared to deal with imminent bacterial infection by reduction of amino acid levels but also by elimination of inorganic and organic nitrogen sources from the apoplast, where pathogens reside during infection.

The strongest regulated gene is glutamine-dependent asparagine synthase 1 (ASN1), which was strongly downregulated during day 3 and day 4 (Table 2). After pathogen infection in Arabidopsis, tomato and pepper, this enzyme has been shown to be upregulated locally^13,39,40^ and its product asparagine is supposed to be important during pathogen infection. Since asparagine serves as a major metabolite in N recycling and translocation^15^, it is suggested that during defense asparagine synthesis by ASN1 may be crucial for the export of nitrogen from locally infected leaves, in order to reduce their nutritional value by limiting nitrogen supply for the pathogens^39,41^.

In detail, Ward *et al*.^13^ found that ASN1 was strongly upregulated after 12 h locally in leaves treated with virulent *Pst*, but not regulated in leaves treated with the hrp^−^- mutant of *Pst* that due to a lack of the TTSS cannot utilize its effector proteins to alter host cell metabolism. Thus, it is suggested that the local upregulation of ASN1 is facilitated by a signalling mechanism that is activated by the pathogen itself in order to gain access to valuable amino acids. Furthermore, glutamate decarboxylase GAD1 is strongly upregulated in leaves treated with the virulent pathogen and to a much lesser degree in hrp^−^ - treated leaves^13^, again suggesting a transcriptional activation directed by the pathogen. This probable pathogen-induced gene activation may in advance be counteracted in systemic leaves to minimize the effects of transcriptional control taken over by a pathogen during an infection.

**Figure 3:**
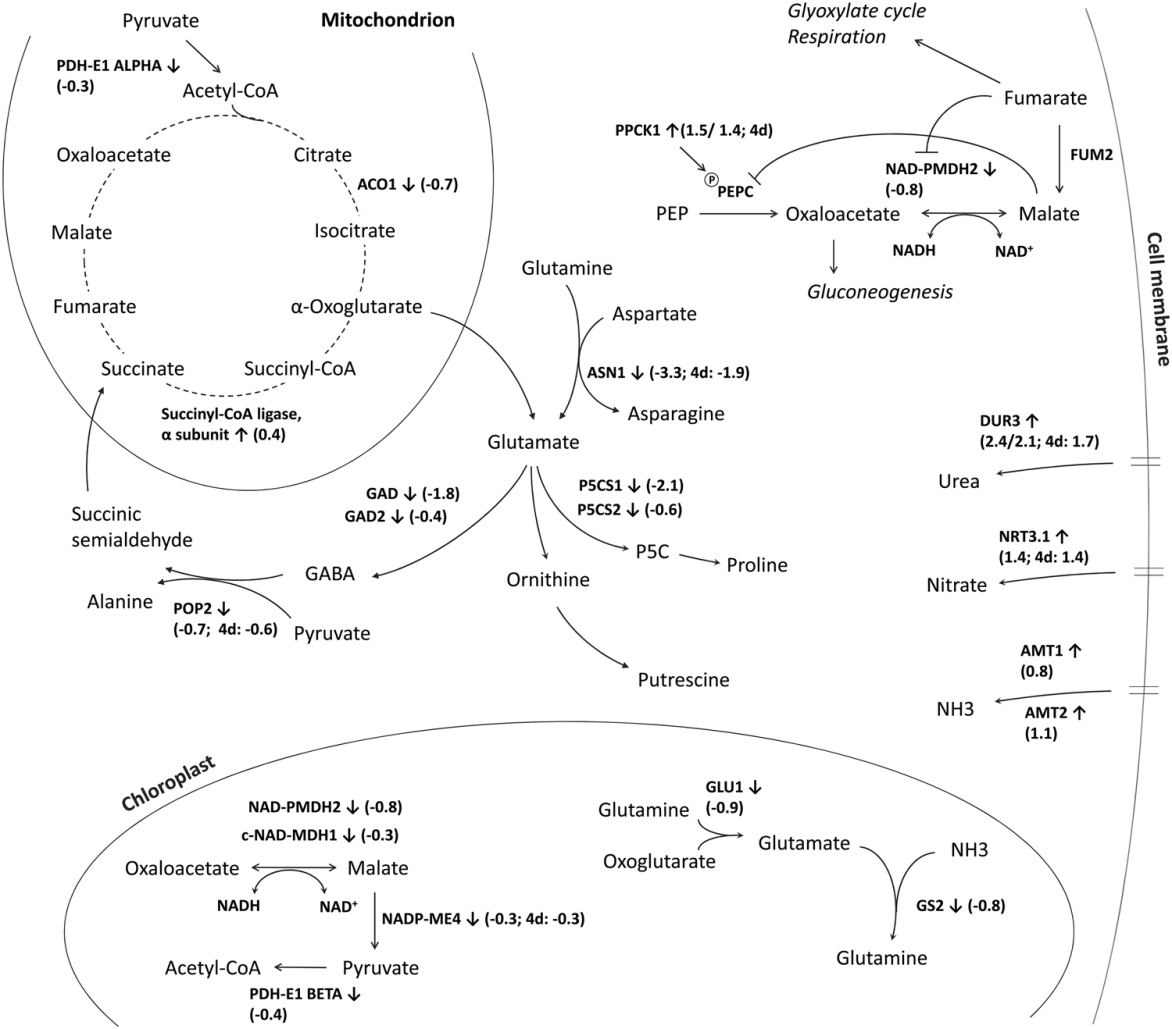
Overview of metabolites and genes related to C and N metabolism that were regulated in systemic leaves. Log_2_-fold changes of gene transcripts are given in brackets for the 10 h time point at day 3 and day 4.

In our experiments, ASN1 was accordingly largely downregulated systemically, together with other major enzymes of N-metabolism, such as glutamate dehydrogenases GAD and GAD2, POP2, glutamate synthase GLU1 and glutamine synthase GS2, as well as several transaminases that interconvert amino acids and organic acids (Table 2, Fig. 3). Apparently central parts of N-metabolism are inversely transcriptionally regulated in systemic compared to locally infected leaves. Moreover, GABA was shown to be largely increased in infected leaves (by a factor of 9 after 18 h; 13). Yu *et al*.^20^ found that *P. syringae* growing in the apoplast strongly expresses genes related to GABA uptake and catabolism, indicating that the pathogen may use GABA as a source for C and N. Thus, reduced levels of GABA in the apoplast would diminish the nutritional value of the apoplastic fluid. Accordingly, in our experiment, three genes of the GABA shunt are downregulated at day 3, glutamate dehydrogenases GAD and GAD2 (lg2fc: −1.8 and −0.4) and POP2 (lg2fc: −0.7) that produces succinic acid semialdehyde, a catabolite of GABA (Fig. 3). Together with the reduced levels of glutamic acid this likely causes reduced GABA levels. Thus we find the hypothesis of reduced N-availability in systemic leaves further substantiated. We attempted to but unfortunately could not detect GABA by our profiling tools to confirm our hypothesis at the metabolic level.

We also found reduced levels of proline (Table 3), which is synthesized from glutamic acid via pyrroline-5-carboxylate (P5C, Fig. 3). In the cytosol, the generation of P5C is regulated by delta 1-pyrroline-5-carboxylate synthase 1 and 2 (P5CS1 and 2), both of which are downregulated at day 3 and back at control levels at day 4 (Table 2). P5C has been associated with the onset of the hypersensitive response during pathogen infection and increases locally 12 h after infection^42,43^. However, the previous focus has been set on the P5C produced in the mitochondrion, which is dependent on P5C dehydrogenase. In our system, we observe again inverse regulation in systemic leaves, namely a downregulation of P5C and proline, as well as glutamic acid. A reduction of several amino acids in systemic leaves was also observed by Wang *et al*.^44^, whereas Návarova *et al*.^45^ did not find reduced amino acid levels. However, these studies were made 2 days after priming where also stronger transcriptional changes were observed^46^ and therefore describe an earlier response where nitrogen metabolism is likely not yet affected.

Together with the systemic upregulation of different N transporters, the down-regulation of N-metabolism in systemic leaves indicates primed limitation of nitrogen availability. This regulation may be a direct effect of signals derived from infected leaves that lead to a reduced import of N into systemic leaves. We can currently not rule out a second mechanism that may be attractive to investigate in the future. Namely, our observations may be indirectly affected by infection-reduced N-uptake through the root system. A possible reason for the latter may be that exported N-containing metabolites from the locally infected leaves^15,41^ may accumulate in the roots rather than, as we show, in systemic leaves. Redistribution to the root would protect valuable metabolite resources from pathogens and may concomitantly reduce N uptake by the root through a negative feedback. It has already been shown that plants can re-allocate N from infected tissues. For example, N transport from root to shoots is increased after root herbivory in *Centaurea maculosa* ^47^, and this is accompanied by a generally reduced N uptake by the root. Further experiments will be necessary to address the systemic N signalling and N distribution along with changes of N fluxes between tissues that are infection-prone and those that are potentially protected or unlikely to be infected by leaf-pathogenic bacteria.

### N limitation is not accompanied by C limitation of systemic leaves

One alternative explanation of limited N metabolism may be decreased availability of carbon and energy in systemic leaves that as a consequence restrict N assimilation. In fact, glucose levels are reduced as well as glycolysis-related genes (Table 3, Supplementary Fig. S1). Similarly, key genes related to starch synthesis, such as ADP-glucose pyrophosphorylase (lg2fc: −0.6), starch synthase 3 (lg2fc: −0.7) and phosphoglucomutase 3 (lg2fc: −0.4) (Table 2) were affected as were photosynthesis-related genes (Table 1, Supplementary Table S3). On the other hand, several senescence-associated genes that are strongly induced under sugar starvation, such as SEN1, BCKDH E1β, ASN1 and MCCA^48^, were downregulated at day 3 and 4, suggesting that there is no limitation in metabolically available sugar (Table 2). Furthermore, sucrose levels were unchanged and especially fumarate and malate levels are largely increased in systemic leaves (Table 3). Therefore, available carbon seems not to be limiting and cannot directly explain reduced N-metabolism.

### Fumarate and malate serve as alternative carbon stores

The strongest effect at the metabolic level is the persistent accumulation of malate and fumarate (Table 3). Both organic acids derive from two different and alternatively localized pathways. First, malate and fumarate are part of the mitochondrial TCA cycle that, however, can operate in different modes. Either the circle operates in a fully closed (complete) mode or it may be open and incompletely used in parts only. The opening of the TCA cycle is induced by an increased redox level under photorespiratory conditions during the day^49^. Under these conditions, one branch of the TCA cycle produces citrate and 2-oxoglutarate, whereas the other produces fumarate and malate that can both be exported from mitochondria and accumulate in the vacuole. The vacuole itself functions as a store or buffer to maintain cytosolic homeostasis of these organic acids. Second, both metabolites can also be synthesized in the cytosol, where malate is generated by the action of PEP-Carboxylase (PEPC), which catalyses CO_2_ fixation via oxaloacetate that is reduced to malate and further converted to fumarate by fumarase (50, Fig. 3). In our study, we found that a PEPC kinase, PPCK1, is upregulated at day 3 (lg2fc: 1.5). PPCK1 is mainly expressed in rosette leaves and the activity of this kinase is directly related to its transcriptional state, which is dependent on light and C supply but independent of N supply^50,51^. PEPC is oppositely regulated at enzymatic level by PPCK1 and malate. Malate inhibits PEPC activity whereas PPCK1 phosphorylation reduces the sensitivity of PEPC towards malate^52^. Accordingly, plants that have reduced PEPC activity produce less malate and increased starch^53^. Champigny and Foyer^54^ suggest the view that phosphorylation of PEPC regulates the co-ordination of nitrogen assimilation, CO_2_ fixation and carbon partitioning by redirecting carbon flow toward the biosynthesis of amino acids. As a consequence we hypothesize that malate accumulation is favoured by a primed desensitizing mechanism of the negative malate-PEPC reaction feedback. In support of our hypothesis, NAD-malate dehydrogenase is also downregulated (lg2fc: −0.8), indicating a reduced conversion of malate to oxaloacetate. Taken together, the transcriptional regulation of enzymes related to the anaplerotic phosphoenolpyruvate pathway may explain the increased biosynthesis of malate and, in turn, fumarate. We assume that the TCA cycle may play a minor role in the primed systemic leaf system.

Fumarate was previously reported to be mainly present in photosynthetically active tissue^55^. Under optimized growth conditions it can accumulate in Arabidopsis leaves to levels that may exceed starch and soluble sugars, such as sucrose, glucose, and fructose^56^. Fumarate is suggested to be required for nitrogen assimilation^57^ and to maintain turgor pressure. More importantly, it can function as an alternative to starch as a flexible storage of photo-synthates that is readily metabolically accessible^58,59^. Accordingly, fumarate-deficient plants with silenced fumarase accumulate twice as much starch in their leaves as wild-type^57^, while starch-less mutants with silenced phosphoglucomutase accumulate up to 5 times more fumarate^56^. These reports suggest that the high primed fumarate levels that we observed in systemic leaves may represent an alternative mode of photo-assimilate storage during ongoing photosynthesis. The accumulated pool of fumarate would be an easily accessible resource if a second infection follows primary local infection in systemic leaves. Fumarate can provide both energy and carbon for rapid and enhanced production of defense responses.

Similarly, malate is an end product of cytosolic glycolysis and the major substrate for mitochondrial respiration. Four malic enzymes, NADP-ME1 to 4, catalyse the reversible decarboxylation of malate. NAPD-ME2 is located in the cytosol and provides most of the total NAPD-ME activity in *Arabidopsis* ^60^. This enzyme may be activated by fumarate under optimized growth conditions and decarboxylates malate to produce pyruvate, CO_2_ and NADPH. However, if fumarate levels are high, NADP-ME2 is strongly inhibited, most likely by competitive inhibition of malate-binding to the active site^58^. The metabolic flux through this enzyme seems to be largely blocked in primed systemic leaves by high fumarate levels that may thereby contribute to concomitantly increased malate levels. Whether the regulation of fumarate and malate metabolism in systemic leaves is a consequence of changes in nitrogen metabolism or whether organic acid accumulation is actively and directly regulated remains to be elucidated.

### Influence of the circadian rhythm

Both metabolic and transcriptional responses are incongruent over the three time points, and apparently large parts of the primed transcriptional responses are attenuated by the circadian rhythm shortly before darkness (Tables 1 and 2). However, in the course of the next day, the majority of primed genes is reactivated. No genes associated with primary metabolism remain regulated shortly before the onset of the night at day 3. This attenuated response shortly before night is still associated with systemic defense priming but other than this core response, it is obviously not maintained at the end of the light phase. Likely plants shut down energetically costly pathways that may be difficult to maintain during the night when no photosynthetic energy production is available. The stronger regulation of gene terms related to RNA metabolism at day 4 indicates that the plant may begin to re-arrange the transcriptional profile to the state before the priming response.

### Abiotic and biotic stress responses

Several jasmonic acid- and ethylene-inducible genes were downregulated in primed leaves (Table 2). The plant defensin genes PDF1.2, 1.2b, 1.2c and 1.3 were strongly downregulated at 3 day/10 h samples, but not regulated at 15 h, and only PDF1.2 was upregulated at day 4. This indicates that defense genes that are associated with jasmonic acid and ethylene signalling are suppressed in bacteria-primed leaves. *Pseudomonas syringae* is known to produce coronatine, which is a mimick of the bioactive hormone conjugate jasmonic acid-isoleucine^61^. JA and SA signalling pathways were previously discussed to negatively interact^62,63^. It is assumed that the bacterial pathogen initiates JA-signalling via coronatine to interfere with the SA-response that is activated immediately after the plant has sensed the pathogen through its PAMPs. However, the downregulation of parts of the JA-elicited defense response that we found in primed systemic leaves suggests that the plant in advance suppresses this signalling pathway, which is potentially activated by pathogens. This primed state thus would sustain an unabated SA-response during the counter-active coronatine signalling during a following pathogen infection. As a disadvantage of this primed state, the plant could be more susceptible to attack by necrotrophic pathogens, what requires JA-based defense responses.

Further, responses to several abiotic stresses were downregulated, such as osmotic, cold, oxidative and salt stress (Table 1), indicating that the salicylic acid-driven response to pathogenic bacteria interferes with signalling related to these abiotic stresses. These effects may have consequences for plants that are under multiple combinatorial biotic and abiotic stresses at the same time and cause negative crosstalk between biotic and abiotic signalling pathways.

## Conclusion

Our results show that systemic alterations of primary metabolism are induced by bacteria infection. These changes are present at the transcriptional and the metabolic level and may determine a priming response in systemic leaves, which is under circadian control. In systemic leaves, levels of nitrogen containing compounds, mainly amino acids, are reduced (Table 3, Fig. 3), as well as transcription of several genes related to amino acid metabolism (Table 2, Fig. 3), leading to an upregulation of genes related to nitrogen uptake of the cell from the apoplast. This is in contrast to the response of locally infected leaves, where amino acids generally increase during the infection^13^. Furthermore, soluble sugars decrease (Table 3) in systematic leaves but apparently do not cause sugar starvation at transcriptional level (Table 2). Rather, carbon accumulates in form of the organic acids fumarate and malate. The re-balancing of nitrogen and carbon in systemic leaves may prime for decreased proliferation of pathogens by limited available nitrogen, whereas increased fumarate and malate may serve as an alternative plant energy source and prime defense responses, if systemic leaves should become infected.

## Electronic supplementary material

Supplementary Figure S1

Supplementary Table S1

Supplementary Table S2

Supplementary Table S3
