# Supplementary material for: Primed primary metabolism in systemic leaves: a functional systems analysis
Source: Sci Rep. 2018 Jan 9;8:216. doi: 10.1038/s41598-017-18397-5 (PMC5760635; doi:10.1038/s41598-017-18397-5)

**Primed primary metabolism in systemic leaves: a functional systems analysis**

Jens Schwachtje, Axel Fischer, Alexander Erban, Joachim Kopka

**Supplementary Figure S1**

Overview of metabolites and genes related to glucose metabolism that were regulated in systemic leaves. Log2-fold changes of genes are given in brackets for the 10 h time point at day 3 and for day 4.


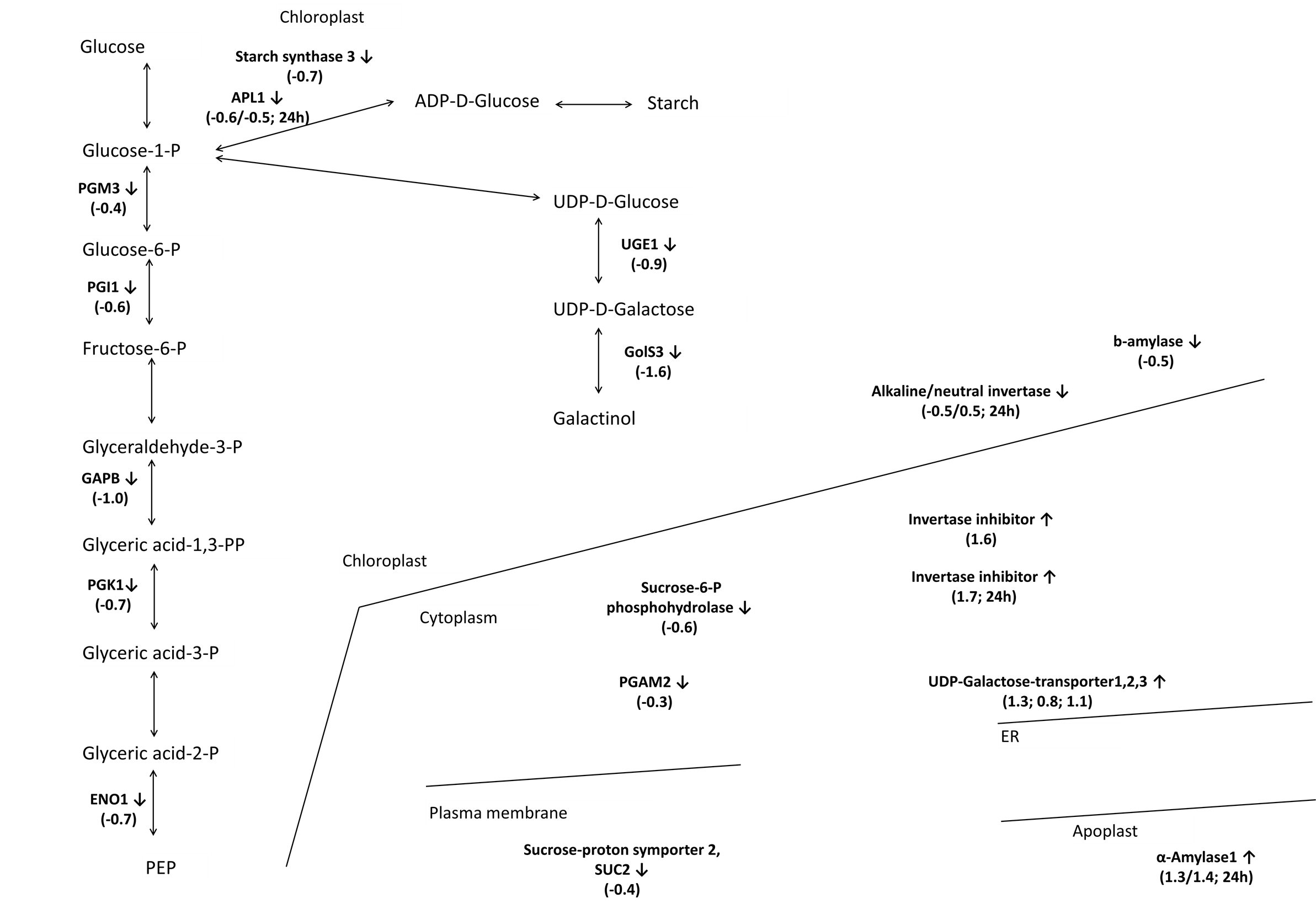

Supplement: Supplementary file 1 — Supplementary Figure S1 [file 41598_2017_18397_MOESM1_ESM.doc]
